# A Systematic Review of the Human Accelerated Regions in Schizophrenia and Related Disorders: Where the Evolutionary and Neurodevelopmental Hypotheses Converge

**DOI:** 10.3390/ijms24043597

**Published:** 2023-02-10

**Authors:** Maria Guardiola-Ripoll, Mar Fatjó-Vilas

**Affiliations:** 1 FIDMAG Germanes Hospitalàries Research Foundation, 08830 Sant Boi de Llobregat, Barcelona, Spain; 2 CIBERSAM (Biomedical Research Network in Mental Health, Instituto de Salud Carlos III), 28029 Madrid, Madrid, Spain; 3 Departament de Biologia Evolutiva, Ecologia i Ciències Ambientals, Universitat de Barcelona, 08028 Barcelona, Barcelona, Spain

**Keywords:** evolution markers, human accelerated regions, HARs, human neurodevelopment, brain configuration, cognitive abilities, schizophrenia, autism

## Abstract

Schizophrenia is a psychiatric disorder that results from genetic and environmental factors interacting and disrupting neurodevelopmental trajectories. Human Accelerated Regions (HARs) are evolutionarily conserved genomic regions that have accumulated human-specific sequence changes. Thus, studies on the impact of HARs in the context of neurodevelopment, as well as with respect to adult brain phenotypes, have increased considerably in the last few years. Through a systematic approach, we aim to offer a comprehensive review of HARs’ role in terms of human brain development, configuration, and cognitive abilities, as well as whether HARs modulate the susceptibility to neurodevelopmental psychiatric disorders such as schizophrenia. First, the evidence in this review highlights HARs’ molecular functions in the context of the neurodevelopmental regulatory genetic machinery. Second, brain phenotypic analyses indicate that HAR genes’ expression spatially correlates with the regions that suffered human-specific cortical expansion, as well as with the regional interactions for synergistic information processing. Lastly, studies based on candidate HAR genes and the global “HARome” variability describe the involvement of these regions in the genetic background of schizophrenia, but also in other neurodevelopmental psychiatric disorders. Overall, the data considered in this review emphasise the crucial role of HARs in human-specific neurodevelopment processes and encourage future research on this evolutionary marker for a better understanding of the genetic basis of schizophrenia and other neurodevelopmental-related psychiatric disorders. Accordingly, HARs emerge as interesting genomic regions that require further study in order to bridge the neurodevelopmental and evolutionary hypotheses in schizophrenia and other related disorders and phenotypes.

## 1. Introduction

Schizophrenia (SCZ) is a complex neuropsychiatric disorder characterised by alterations in perception and behaviour. Although the specific aetiological and pathophysiological mechanisms have not yet been fully elucidated, the amount of evidence regarding environmental and genetic risk factors converging in neurodevelopmental pathways [1,2,3,4,5,6,7], has led to the neurodevelopment hypothesis of the origin of the disorder and its posterior revisions. Currently, this hypothesis states that the joint and interacting effect of the polygenic background and environmental stressors disrupt the neurodevelopment and brain maturation trajectories and underlie the later emergence of schizophrenia [8,9,10,11].

It has been proposed that, depending on the neurodevelopmental patterns and the resulting brain functional deviations, different mental disorders may emerge [12]. Apart from the shared symptoms, there is also an important genetic overlap across psychiatric disorders, which converges in common biological pathways related to transcriptomic regulation and synaptic plasticity [12,13,14,15,16]. These commonalities in behavioural patterns and biological pathways have led to the placement of different mental disorders—such as schizophrenia, other psychotic disorders, and autism spectrum disorders—along the same continuum [12].

Genetic diseases and disorders that arise from the cumulative effect of many genetic variants are thought to persist in human populations due to a subtle balance between mutation, genetic drift, and natural selection. Thus, the alleles conferring risk are introduced in the population by mutation, persist due to random genetic drift, and are eventually eliminated by purifying selection [17,18,19,20]. The epidemiological characteristics of SCZ—such as its onset in late adolescence or early adulthood and the reduced fitness presented by affected patients, especially in males [17,20]—should lead to the eventual removal of genetic risk factors from the genetic pool. Having said this, SCZ is still a relatively common disorder with a similar prevalence across countries and regions whereby it is estimated to be present in 0.28% of the global human population [21]. The world-wide homogeneous SCZ prevalence [21] is also related to the question of when the genetic liability for SCZ emerged. In this regard, genome-wide association studies (GWAS) in SCZ that were conducted in African, Asian, and European populations identify the involvement of mostly the same genes and pathways that are related to neurodevelopment [2,22,23]. This implies that the genetic variability underlying SCZ is common in all current human populations, and that evolutionary changes underlying the emergence of neurodevelopmental human specificities would probably precede the divergence of human populations.

Therefore, the next question is why the genes that increase the likelihood of suffering from SCZ have persisted in the human genome. This evolutionary paradox has been discussed through various models and evolutionary mechanisms, such as balancing selection, fitness trade-offs, fluctuating environments, sexual selection, mutation–selection balance, and background selection [24,25,26,27]. While the details have been reported elsewhere [26,27], these evolutionary scenarios are not mutually exclusive and converge in the idea that SCZ’s genetic underpinnings emerged as a costly by-product in relation to the evolution of the ontogenetic mechanisms sustaining human-specific neurodevelopment and higher-order cognitive abilities [28,29,30].

It is believed that the expansion of the cortex during primate evolution, especially in terms of the human lineage, has contributed to the assembly of more complex neuroarchitectures, which are able to withstand higher cognitive abilities—such as abstraction, language, memory, attention, awareness and thought [31,32,33,34,35]. Although the neurodevelopmental patterns, cytoarchitecture, cell type composition, and neurogenic gene expression programs of humans, macaques, and chimpanzees are remarkably similar [36,37], the genes involved in the respective neurodevelopmental trajectories exhibit tempo-spatial expression differences between humans and chimpanzees [36]. Additionally, human and macaque culture assays point towards an environmental independence of the progenitor neurons’ developmental timing [35], thus suggesting that the corresponding neurodevelopmental processes (and particularly neurodevelopmental timings) are highly genetically determined.

Therefore, the study of human-specific genomic changes as compared to our closest living relatives may lead to a better comprehension of human-specific phenotypic traits [38]. In this sense, the results indicate that human particularities could be intimately related to non-coding DNA variability, thus leading to a differential control of the transcriptional networks [39]. Indeed, comparative expression studies describe differences in gene expression patterns between humans and primates during critical neurodevelopmental periods in regions such as the prefrontal cortex [36,40].

To identify the evolutionarily relevant genomic regions that are responsible for these differences, it is possible to take advantage of comparative genomics, which enables the identification of genomic loci that are highly divergent between different species. Based on this idea, Pollard et al. [41] identified the Human Accelerated Regions (HARs). HARs are evolutionarily conserved genomic elements across mammals’ evolution that have rapidly accumulated human-specific DNA sequence changes since the divergence from the human–chimpanzee ancestor. After the first discovery of HARs, several studies described other HAR sets based on different methodologies [42,43,44,45,46]. Nonetheless, the accelerated divergence of HARs between humans and their ancestors is suggested to reflect their role in human evolution, as well as in respect of their association with certain human-specific traits [47,48,49], such as neurodevelopment mechanisms and outcomes [50]. Most of these HARs do not code for proteins and are in intergenic regions or within introns near protein-coding genes, transcription factors, and DNA binding proteins, thus pointing towards their role as regulatory elements and RNA genes [41,50,51].

Indeed, HARs’ role appears to be mediated through their predicted function as developmental enhancers, whereby some of them operate with human-specific activity [50]. For example, the enhancer activity testing of several non-coding HARs drew attention to HAR238, which showed spatial activity differences between humans and chimpanzees during forebrain development. Such inter-species tissue-differential expression patterns are proposed to be responsible of the changes in the enhancer effect of HAR238 on the nearby gene *GLI2* [50], a zinc-finger transcription factor in the Sonic Hedgehog signalling pathway that is critical for the induction of neural tube formation. These results guide the view of how small sequence changes in HARs may modify the complex patterns of gene expression that are necessary for proper development in a human-specific manner.

All the HARs in a person are estimated to contain an average of 1273 genetic variants and have a two-fold depletion of rare variants when compared to coding, noncoding conserved, and randomly selected loci [52]. Thus, the variants that endowed HARs their human specificity are not the same that provide human variability because the human-specific sites account for only around 2% of HAR variants [52]. Moreover, comparative genomics data suggest that the rare mutations in HARs are generally deleterious, which is reflected in a paucity of recent alleles (non-ancestral alleles 8.3%) and the fixation of 96% of ancestral alleles [52,53]. As such, some authors have suggested that HARs could have gone from positive selection along human evolution to a possible switch back to negative selection within human populations [53].

Inspecting the role of HARs and the associated genes could provide novel insights into the human brain’s uniqueness and the pathogenesis of mental disorders. Although there are reviews on HARs’ role in the genome, these are mainly focused on their role during neurodevelopment [54,55,56,57]. Moreover, in the past two years, HAR-based studies on human-specific brain traits and psychiatric disorders have been a burning issue. In addition, there is a need to review the role they play in human brain configuration, particularities, cognitive abilities, and neurodevelopmental-related psychiatric disorders. Accordingly, we aimed to conduct a systematic review of the literature with respect to HARs’ role in neurodevelopment, brain structure and function, cognition, and psychiatric disorders susceptibility. 

Starting with evidence regarding HARs’ biological function as neurodevelopmental regulatory elements, we continue to review their modulatory role in the context of human-specific brain configuration, able to sustain higher-order cognitive abilities, such as intelligence or sociability. Lastly, we summarise the findings that directly relate HARs’ genetic variability with SCZ, as well as other neurodevelopment-related disorders and syndromes. Therefore, our ultimate objective is to join data in order to evaluate whether the study of these evolutionary markers could help to shed light on the understanding of the genetic component of neurodevelopment and major psychiatric disorders.

## 2. Methods

We conducted a systematic search from April 2021 to November 2022 in the PubMed and Web of Science (Web of Science Core Collection) databases, searching for articles from inception to 14th November 2022. For the search strategy, we used the following terms: (“human accelerated regions” or “accelerated regions” or “accelerated gene” or “human accelerated genes”) and (“schizophrenia” or “psychiatric disorder” or “neurodevelopment” or “brain development” or “development” or “cognition”). The inclusion criteria were English-written peer-reviewed original articles and studies that aimed to assess the role of Human Accelerated Regions, or their related genes, on neurodevelopment, brain-based phenotypes, or psychiatric disorders. The studies that did not fulfil these criteria were excluded.

The papers were initially screened based on the title and abstract. Those meeting the inclusion criteria were analysed by the two researchers who independently performed the data extraction using a form. This form included the main article aims, the HARs approach (candidate vs. whole-genome), the main methodology, findings, strengths and limitations, and the classification of the article according to three major topics (HARs in neurodevelopment, HARs in the brain and cognitive phenotypes, and HARs in psychiatric disorders). The Preferred Reporting Items for Systematic Reviews and Meta-analyses (PRISMA) guideline was followed to guarantee the good quality of the systematic review.

## 3. Results

### 3.1. Literature Search and Study Selection

**Figure 1:**
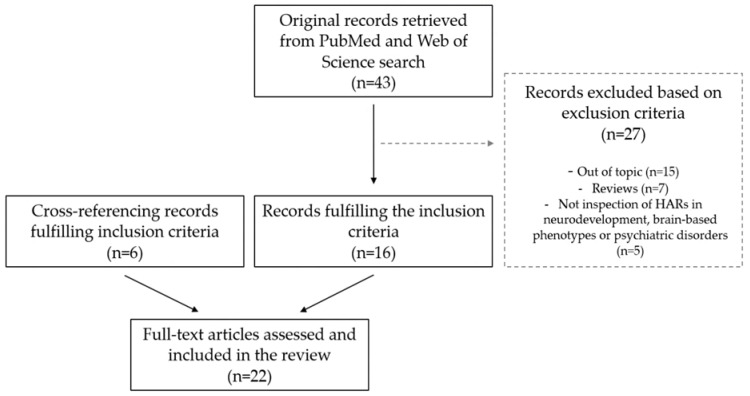
Flow diagram of the literature search and article selection.

Following the systematic search strategy and study selection, 16 publications that met the inclusion criteria were included. Additionally, six articles that also met the inclusion criteria were found from cross-referencing and were additionally reviewed. Thus, a total of 22 articles were considered in the final qualitative synthesis (Figure 1).

A summary of the main findings is presented around three different topics. The first topic encompasses the role of HARs during neurodevelopment. This includes the articles that have assessed the role of HARs and the nearby and interacting genes (HAR genes) during neurodevelopment. This was achieved using genome-wide approaches to conduct histone profiling, chromatin conformation data, DNase I sensitivity, and massively parallel reporter assays [52,58,59,60] (Table 1), and studies that assessed the role of several candidate HARs and genes associated with HARs (HAR genes) concerning their neurodevelopmental expression [47,52,58,60,61,62,63,64,65] (Table 2). The second topic includes research on the role of HARs in the context of adult brain structure, function, and cognitive and behavioural phenotypes [66,67,68,69] (Table 3). Lastly, the third topic comprises research on HARs’ involvement in psychiatric disorders. Most of the studies are focused on SCZ [66,69,70,71,72,73,74,75,76], but there is also research on other neurodevelopmental psychiatric disorders and phenotypes, such as autism spectrum disorders (ASD) [52,58,66], bipolar disorder [76], and delirium syndrome [77] (Table 4).

### 3.2. HARs and Neurodevelopment

#### 3.2.1. HARs’ Function in Neurodevelopment

The first attempts to uncover the role of HARs during neurodevelopment from a genome-wide perspective were through in silico tools (Table 1). These were designed to search for specific genomic motifs that are associated with histone marks, active chromatin, and transcription factor binding sites (TFBS).

First, it was pointed out that HARs showed significant enrichment for foetal and adult brain histone profiles. These histone profiles were (i) accessible in neurogenic zones of the developing cortex and (ii) specific to neural progenitor cells and maturing neurons, thereby suggesting their activity both in the developing and adult brain [52,58,60]. Analyses indicated that >75% of HARs possessed marks of active chromatin states, of which more than 45% were active in neural and foetal brain tissue [52,58,60]. On the one hand, HAR regions were enriched in TFBS (DNA regions where transcription factors bind to promote transcription). Indeed, many of them were associated with transcription factors specifically involved in neurodevelopment (e.g., *MEF2A*, *SOX2*, *ZNF333*, *REST*, *CTCF,* and *NFIA* [52]) and with roles during cortical neurogenesis (e.g., *RBPJ*, *TBR2*, *PAX6*, *ATOH1,* and *TED* [60]). In this regard, the HAR sequence comparison between humans and chimpanzees suggested that the human-specific genetic changes in HARs altered the TFBS motifs (with losses or gains of them), thus pointing towards species-differential regulation of neurodevelopmental processes [52]. On the other hand, specific assays evidenced the role of certain HARs as transcription factors per se, with a precisely regulated activity during the different neurodevelopmental periods. For example, certain HARs with enhancer activity in neural progenitor cells were enriched for transcription factor (TF) motifs belonging to cortical patterning and cell maintenance. Other HARs active in maturing neurons presented TF motifs associated with cell fate determination and cell differentiation [60].

Later, massively parallel reporter assays (MPRAs) and capture MPRAs (CaMPRA), which allow targeting HARs to evaluate their enhancer activity in vitro, have contributed to validating in silico predictions. These analyses evidenced that between 13% and 49% of HARs possessed enhancer activity in human neural cells. Additionally, they showed that between 28% and 61% of HARs presented different enhancer activity in humans when compared to chimpanzees [59,60]. While further data are needed, these HAR orthologs comparisons indicated that human-specific HAR sequence changes were mostly associated with increased enhancer activity in humans [60]. Interestingly, those HARs that presented differential activity between humans and chimpanzees overlapped with regions with different chromatin accessibility. This, therefore, suggested that human-specific sequence changes could modify chromatin conformation and accessibility, thereby producing changes in the enhancer activity [59].

#### 3.2.2. Genes Associated with HARs and Their Expression and Functional Patterns

The genes associated with HARs (HAR genes) have been defined according to different methodologies, such as positional mapping, proximity, or functional interaction (Table 1). Positional mapping has allowed the identification of genes that include HARs within their introns or near the 5′ and 3′ UTRs. Other HAR genes, however, have been retrieved from chromatin interaction data and chromosome conformation capture [52,58]. The biological processes and pathways in which these HAR genes participate include neurodevelopmental processes, neuronal differentiation, and axonogenesis [52].

Chromatin interaction analyses in human fibroblasts evidenced that many HARs interacted with the promoters of the flanking genes and allowed the mapping of 21% of HARs to 700 target genes [52]. Nonetheless, chromatin interactions are predicted to be of high tissue- and cell-type specificity, thus suggesting that more accurate methods for HAR gene identification are required. In this sense, with the use of three-dimensional chromatin interaction data in the developing human cortex, Won et al. [58] were able to map 38% of HARs to 1648 genes that were active in the cortical plate and the germinal zone. These results again evidenced the fact that HAR genes were related to neurodevelopmental pathways. Moreover, more specific biological processes emerged, such as neural regionalisation, dorsal–ventral patterning, cortical lamination, and the proliferation of neuronal progenitors. This approach indicated that while proximity can be a good indicator of the target genes, it should not be considered the only factor. This is important since the interactions between HARs and genes are cell-type-specific. Additionally, it was described that HAR genes (such as *SOX2*, *PAX6*, *POU3F2*, *GLI3*, *EN1,* and *TBR2*) played major roles in cerebral cortex development and dorsal–ventral/anterior–posterior pattern specification [58]. Convergently, data from chromosome conformation in neural progenitor cells and human neocortical stem cells also implicated HAR genes in functions related to cell differentiation and development [59].

From a functional point of view, HAR genes identified through chromatin interaction data presented elevated dosage sensitivity levels (defined as a significantly elevated haploinsufficiency), especially in those that were neurally active, when compared to the rest of the genes in the genome. This trait could suggest that their pathogenicity may be mediated by alterations in the expression levels [52]. In relation to this, HAR genes present loss-of-function intolerance levels that are typical of genes undergoing strong purifying selection [58]. Following on from this, the expression modulation role of HARs has been conceptualised as a human-specific source of dosage regulation [52].

Expression analyses across developmental stages evidenced that HAR genes were highly expressed during prenatal development and sharply upregulated during neurogenesis—peaking near mid-gestation, which is a period marked by neuronal migration, early neuronal phenotype definition and dendritic arborisation [58]. In addition, HAR genes were predominately expressed in cells from the outer radial glia, which is a major class of neural stem cells in the germinal layer that shows substantial expansion in the primate lineage [58]. Analyses in adult prefrontal cortex samples suggested that HAR gene expression was enriched in neuronal cell types, rather than in glia. Additionally, HAR gene expression was enriched in superficial cortical layers, which form the inter- and intra-hemispheric connections between cortical regions and are significantly expanded in primates [58].

#### 3.2.3. Regulatory Effect of Candidate HARs on Their Proximal Genes

Complementary to whole-genome HAR analyses, different experiments have focused on validating the regulatory activity of specific HARs on candidate genes and have contributed to unravelling their role during neurodevelopment (Table 2).

While the functional analyses were conducted using different methodologies, as well as in cellular or animal models, all candidate HARs analysed thus far are expressed in neural-like cell types [47,52,58,60,61,62,63,64]. Interestingly, HAR1 was found to be part of two previously unknown RNA genes, *HAR1F* and *HAR1R,* which are expressed in the foetal and adult human brains, albeit with still unknown functions. In the foetal brain, *HAR1F* (also known as *HAR1A*) is expressed in the neocortex, specifically in Cajal–Retzius neurons and co-detected with reelin; meanwhile, *HAR1R* (also known as *HAR1B*) expression is less localised and intense. In the adult brain, *HAR1F* is expressed in the frontal cortex, the hippocampus, the thalamus, and the hypothalamus, while, again, *HAR1R* expression is blunted [47].

The vast majority of the 29 HARs validated operate as transcriptional enhancers of genes related to neurodevelopmental processes [52,58,60,62,63,65]. Only one HAR, HACNS174, associated with the *AUTS2* gene, did not evidence conclusive enhancer function [64]. Meanwhile, two other HARs performed distinct roles: HAR1 was an integral part of two RNA genes [47], and the HAR flanking the *HSTR1* gene functioned as its promoter [61].

From a functional point of view, the analysed HARs were found to interact with genes that perform crucial roles for brain development and wiring, such as being associated with developmental signalling pathways and homeostasis maintenance, such as *NPAS3*, *AUTS2*, and *GLI2* [62,63,64]; being related to neuronal differentiation and proliferation, such as *PTBP2* or *PPP1R17* [52,60]; and being implicated in synaptic and cortical development, such as *FZD8*, *CUX1*, *GPC4*, *GLI3*, and *TBR1* [52,58,65]. Indeed, genes such as *PPP1R17*, which regulates the neural progenitor cell cycle progression, as well as the slowing down and lengthening of the neural progenitor cell cycle, are of particular interest for human-specific cortical development. As described by Girskis et al. [60], the *PPP1R17* expression in humans was restricted to foetal development, driven by cortical neural progenitor cells in contraposition to the macaques’ expression, which continued in adulthood in cortical astrocytes.

The studies that evaluated the effect of HARs’ human-specific substitutions describe that those genetic changes that had functional consequences were related either to higher promoter activity [61] or to a gain or loss of transcription factor binding sites [62,63,65]. Additionally, the studies that compared HAR ortholog sequences among humans, primates, and mice also reported human expression differences during neurodevelopment compared to the other species [61,62,63,65]. For instance, analyses in transgenic mice evidenced that the human-specific substitutions in the enhancer HARE5 associated with the *FZD8* gene produced faster cell cycles when compared to the HARE5 ortholog sequences in chimpanzees and mice. Furthermore, this, in turn, resulted in greater cortical gyrification and larger mice cortices [65]. Moreover, transgenic mice expression analyses also showed that while both the human and chimpanzee HARE5 orthologs were expressed in the developing telencephalon, human HARE5 expression occurred earlier and more intensely [65].

### 3.3. HARs and Brain and Cognitive Phenotypes

If HARs participate in the neurodevelopmental gene expression machinery, it appears obvious to investigate their involvement in shaping brain architecture and the cognitive traits derived from it (Table 3).

Under this rationale, after describing that the frontoparietal and the default mode networks were the cortical networks that experienced the larger expansion in humans when compared to chimpanzees, Wei et al. [66] analysed the implication of HAR genes. First, they showed that the spatial expression trajectories of HAR genes and the patterns of cortical expansion positively correlated. Second, the regions of higher-order cognitive networks with the larger expansion in humans (which included the frontoparietal, ventral–attentional, and default mode networks) were also the same networks with the higher expression of HAR genes. With respect to this, the highest correlation was observed within regions of the default mode network (DMN). These results remained significant even when the HAR gene set was limited to those specifically related to brain processes (HAR brain genes) [66]. Indeed, the comparative expression analyses in humans, chimpanzees, and macaques evidenced the fact that the elevated expression of HAR brain genes observed in higher-order cognitive networks in humans was not found to be as high in chimpanzees and macaques. This points towards the fact that, in humans, HAR genes are upregulated in brain areas related to higher-order cognitive functions. Additionally, functional magnetic resonance imaging (fMRI) analyses in healthy subjects revealed that the genetic variability of HAR brain genes affected the functional modulation of the DMN, in contrast to the results in other functional networks [66].

The use of HAR genes and HAR brain genes expression patterns to unravel the genetic determinants of other brain phenotypes is a strategy followed onward in other studies. On the one hand, a study investigating individual differences in functional connectivity described that the highest functional variability across individuals was observed within higher-order cognitive modules such as the frontoparietal network, the dorsal–ventral attentional network, and the DMN [67]. Subsequent analyses on how functional connectivity spatial variability covaried with gene expression patterns revealed that HAR brain gene expression positively correlates with functional variability. An increasing HAR brain gene expression was described from the subcortical regions and primary areas (which showed the lowest individual variability) to the association cortices (with the highest individual variability and the highest HAR expression levels). Actually, HAR brain gene expression accounted for up to 31% of functional connectivity interindividual variability. In line with the findings from Wei et al. [66], HAR brain gene expression was found to be higher in the DMN, dorsal–ventral attentional network, and frontoparietal network. In addition, the most correlated genes were related to the development of the synapses, neurogenesis, and neuron differentiation.

On the other hand, in a cutting-edge study that utilised multimodal neuroimaging analyses and information decomposition methods, Luppi et al. [68] studied the brain’s neural information processing in terms of redundant (or shared information, which provides robustness to the system) and synergistic (or complementary information, which provides integration to the system) brain interactions. Their analyses evidenced that synergistic and redundant brain interactions showed a regional gradient, whereby the redundant patterns were prominent in the brain’s somatomotor, salience subnetworks, and visual regions. Meanwhile, the synergy was predominant in higher-order association cortices that were affiliated with the DMN, the frontoparietal network, and the limbic subnetwork. Human and macaque comparisons highlighted high evolutionary stability but also showed the exceptionality of the prefrontal cortex, which was a synergy-dominated region in humans. This high human prefrontal cortex synergy also correlated with the human-specific cortical expansion previously reported [66], thus suggesting that the additional cortical tissue in humans may be dedicated to synergistic rather than redundant interactions. However, the relevance of these findings for our matter comes when the authors described that the regional predominance of synergy correlated with the regional expression of HAR brain genes, which explained up to 30% of the regional synergy–redundancy variance.

Regarding cognitive phenotypes, the study by Wei et al. [66] took advantage of GWAS data to investigate HARs’ implications in cognitive and social abilities. First, gene-set analyses of HAR genes and HAR brain genes evidenced their association with intelligence. Second, the same gene-set effects modulated a proxy of sociability, which derived from a single question evaluating the frequency of friend and family visits. Also, using intelligence GWAS data, Cheung et al. [69] described that the variability within HAR genes was more likely to be associated with intelligence than SNPs not affiliated with HARs. Additionally, thanks to brain gene expression data across neurodevelopmental stages (from early prenatal to adulthood), their findings highlighted that HAR genes highly expressed in the brain across the different neurodevelopmental stages are associated with intelligence.

### 3.4. HARs and Psychiatric Disorders

#### 3.4.1. HARs in Schizophrenia

While using different methodological frameworks, many attempts to investigate HARs’ link with susceptibility to SCZ have been conducted through enrichment analyses. Enrichment methodologies assess whether SNPs associated with SCZ significantly cluster in HAR regions [73], HAR genes [69,71], and HAR brain genes [69] (Table 4).

First, Xu et al. [63] compared the overlap between the GWAS loci that were associated with SCZ, and the genes associated (within 100kb flanking regions) with recently evolved HARs. These recently evolved HARs, named pHARs, were defined based on conservation among non-human primates. Moreover, other ancient accelerated regions, PARs (primate accelerated regions based on conservation among non-primate mammals), and mHARs (based on the conservation among non-human mammals) were investigated. The results indicated that all the SNPs associated with the disorder at *p*-value < 1 × 10^−7^ were significantly enriched within the genes in pHARs as compared to PARs and mHARs. This means that pHARs harboured more SCZ-associated SNPs than would be expected by chance. Subsequent pathway and biological processes analyses in pHARs highlighted the involvement of GABAergic pathways, which, as pointed out by the authors, have been consistently described as dysregulated in SCZ [78]. Moreover, pHAR variability converged in genes related to neuron differentiation, cell adhesion, plasma membrane, and cadherin binding, categories related to brain development and synapse formation, following previous findings [52,58]. Network analysis on gene expression profiles from the human cortex revealed that the pHAR genes associated with SCZ were more connected than other SCZ genes. This, therefore, entails the fact that these pHAR genes were hub genes in the human cortex expression network [71].

Second, Srinivasan et al. [65], through fold enrichment plots, also showed that SNPs within HARs (and in linkage disequilibrium with them) were enriched in susceptibility variants for SCZ. The same analysis but specifically focused on HAR brain genes delivered the same conclusion.

Third, Cheung et al. [69] also evidenced that the genetic variability within HAR genes was significantly enriched with respect to associations with SCZ. In addition, the HAR genes highly expressed in the brain across different neurodevelopmental stages were also associated with this disorder. These enrichment findings would align with previous gene-set results demonstrating that HAR genes and HAR brain genes were associated with genetic variants underlying SCZ [66].

Beyond HAR enrichment findings through genome-wide approaches, there are also data linking subsets of HARs with SCZ. Novel open reading frames (nORF) are genomic loci able to encode for uncharacterised transcripts and protein products. A slight overlap between nORF regions and HARs has recently been described. Some of these overlapping regions have been found to harbour loci associated with SCZ and bipolar disorder through GWAS studies [76]. Furthermore, focusing on the nORFs differentially expressed between healthy controls and patients with SCZ, a HAR overlap was also described. As such, these findings relate HARs with novel sources of transcription and with altered expression in SCZ.

Lastly, candidate HAR genes have also been investigated in SCZ susceptibility through association studies in European and Indian populations. While common and rare single nucleotide variants in the *HAR1A* and *NPAS3* genes were not significantly associated with the risk for SCZ [70,72] in European samples, a six-SNP haplotype in *HAR1F* was associated with the presence of auditory hallucinations in SCZ patients [70]. Conversely, four SNPs in candidate HARs were significantly associated with the disorder’s risk in two independent case–control samples from a north-Indian origin [75]. Additionally, 15 SNPs significantly modulated cognitive performance within either controls or patients from a subsample with cognitive assessments including attention, spatial and working memory, sensory-motor and emotional processing, and abstract and mental flexibility [74]. Nearly all the associated SNPs interfered with TFBS and possessed methylation marks of active promoters, repressors, or enhancers in different brain regions [74,75].

#### 3.4.2. HARs in Other Neurodevelopment-Related Psychiatric Disorders and Syndromes

Although most of the genetic association and enrichment studies of HARs have been conducted in SCZ, if we consider the etiological and pathophysiological continuum across different psychiatric disorders [12] it is inevitable to investigate the role of HARs in other neurodevelopment-related disorders that have previously shown certain genetic and symptomatic overlap with SCZ (Table 4).

In contrast to SCZ studies, the analyses of HARs on ASD have been mainly focused on rare and de novo variability [52,58,66]. When using a sample of 2100 sibling–ASD dyads, Doan et al. [52] observed that rare, previously unidentified (de novo) copy number variants containing HARs were more frequent in ASD probands than in healthy siblings. Afterwards, the impact of mutations in HARs was assessed in 218 consanguineous ASD families (first-cousin marriages, with higher ASD genetic load). In addition, the sequencing of the HARome revealed that affected individuals presented a significant excess of rare mutations, which remained significant even when the analysis was limited to neurally active HARs. These results converge with findings from studies examining the enrichment of HAR genes and HAR brain genes in rare, damaging, and de novo variants. Not only were the rare variants associated with ASD more prevalent within HAR genes and HAR brain genes [66], but rare de novo loss-of-function mutations associated with ASD and developmental delay were also more prevalent within HAR genes [58]. In parallel, analyses on the functional impact of several HAR mutations in ASD-affected individuals through MPRA assays in mouse embryonic cortex neurospheres revealed a higher prevalence of mutations in conserved loci with predicted regulatory function when compared to non-conserved loci. This suggests that variability in HARs may contribute to altering pathophysiological mechanisms in ASD [52].

HAR genes have also been associated with brain structural changes across psychiatric disorders. In particular, it was described that HAR brain gene expression correlated with cortical volume changes that were found across SCZ, ASD, bipolar disorder, major depression disorder, and obsessive-compulsive disorder [66].

Finally, HAR brain genes have been associated with delirium, which is a clinical syndrome characterised by fluctuating disturbances in attention, consciousness, and cognition [79,80]. Through protein–protein interaction network analyses that were used to prioritise genetic pathways associated with a certain condition, it was observed that HAR brain genes were hub genes and among the top network modules associated with delirium [77].

## 4. Discussion

The present review offers an overview with respect to the role of an evolutionary marker, the HARs, as gene expression regulatory elements during human neurodevelopment. This role is supported by results not only showing HARs’ impact in the context of brain architectural and functional configuration of human-specific traits, but also in the inherent cognitive and behavioural human diversity. Also, data on the association of genetic variants in HARs with the vulnerability for schizophrenia and other psychiatric disorders strengthen the idea that HARs may contribute to brain development and function, thus eventually leading to mental disorders.

Recalling the findings on the role of HARs in neurodevelopment, many pieces of evidence converge on the idea that HARs possess paramount roles—presumably as enhancers, but also as other gene expression modulators—concerning genes that guide processes, such as neural proliferation and differentiation [52,58,59,60]. Nonetheless, not all HAR sequences have been functionally validated and thus, unequivocal conclusions are still missing. In addition, as seen by the temporal and tissue specificity of HAR expression [52,58,60], it has been possible to infer the temporal window in which HARs operate, the early neurodevelopment, and the tight expression regulation to which they are subject. Still, as previously suggested [50,81], new research is required to assess the role of HARs in other developmental tissues, stages, and in their native environment.

Focusing on the mechanisms by which HARs exert their effects, certain findings indicate that HARs potentially act as binding sequences of transcription factors that are specifically involved in neurodevelopment and cortical neurogenesis. However, they may also function as transcription factors per se, which are related to cortical patterning, cell fate determination, maintenance, and differentiation [52,60]. Likewise, the fact that between 30 and 60% of HARs show enhancer activity differences between humans and chimpanzees seems to indicate how HARs would have contributed to the human neurodevelopmental uniqueness. Indeed, human-specific substitutions in HARs would change the transcription factor binding landscape [59,60] and would lead, in turn, to changes in gene expression timing, intensity, and patterns and cell cycle lengthening. These expression changes would even result in major phenotypic changes, such as greater cortical gyrification and enlargement, because certain studies on HAR gene expression profiles show such effects [58,66]. Such evidence reinforces the view of the HARome as a contributor to the emergence of human-specific traits and underlying human behavioural diversity.

Beyond the human interindividual variability associated with HARs, there is evidence highlighting their role in human-specific disorders intimately related to brain and conduct traits. Among the genetic determinants shared across neurodevelopmental psychiatric disorders such as SCZ and ASD, the genomic regions that comprise transcription factors regulatory sites stand out, identifying them as a genetic mechanism of interest [82]. In addition, it is remarkable that these regulatory elements are described as potential coregulators of genes that are related to nervous system development, transcription, and synaptic transmission [82]. Therefore, by their neurodevelopmental guidance role, HARs may harbour common genetic determinants shared between different brain phenotypes and psychiatric disorders.

Moving on to the studies directly inspecting HARs’ role with respect to brain phenotypes, all results describe correlations between HAR expression and (i) the human organisation of higher-order cognitive networks [66], (ii) individual functional connectivity variability [67], and (iii) the brain’s synergistic and redundant information processing patterns [68]. These findings point towards the HARs’ involvement in the cortex structural organisation, brain information integration and, ultimately, the brain’s functional and cognitive responses. Indeed, the functional networks highlighted by the different studies have been described not only to be altered in psychiatric disorders [83,84,85,86], but also to sustain complex cognitive processes and social cognition [87,88]. Indeed, these are core traits of humankind [89] that are, in turn, commonly compromised in SCZ and other psychiatric disorders such as ASD [90,91]. Also, we should also consider the results on human-specific brain network organization and its association with brain dysconnectivity in SCZ [92]. It was evidenced that the transcription profile of HAR genes significantly correlated with cortical areas that display human-specific connections when compared to chimpanzee connection features. In addition, the identified human-specific cortical connections mirrored the regions where SCZ patients presented lower fractional anisotropy [92]. Therefore, indirectly, these observations add to the key role of HARs in shaping the architecture of these networks and sustaining human cognitive abilities and the alterations in SCZ and other neurodevelopmental psychiatric disorders.

As is the case with investigations on the association of HARs with psychiatric phenotypes—while genome-wide based studies have consistently reported an enrichment of SCZ-associated variants in HAR regions [52,58,66,71,73]—the candidate HAR approaches have shown less consistent findings [70,72,74,75]. Conversely, other studies have successfully highlighted the role of candidate HARs as susceptibility loci for SCZ and other mental disorders, as is the case of the *NPAS3* [93,94,95]. In line with this, the *AUTS2* gene has been associated with ASD and other disorders, such as attention deficit hyperactivity disorder, epilepsy, and dyslexia [96,97,98]. Thus, future association studies would greatly benefit from the selection of different candidate HARs, such as those that function as neurodevelopmental enhancers of the *AUTS2*, *CUX1*, *GPC4*, *GLI2*, *GLI3*, and *TBR1* genes, which have been related to the biological roots of ASD and SCZ [52,93,99,100,101,102,103]. However, apart from the implication of common genetic variability in the susceptibility for these disorders, there are also data directly involving rare and more penetrant variants [52,58,66]. Independently of the frequency of the genetic determinants, there appears to be a shared HAR-related genetic variability underlying psychiatric disorders, especially SCZ and ASD. As studies on the shared symptomatology and genetic variability suggest [13], if we understand neuropsychiatric disorders in a neurodevelopmental continuum [12], rather than as discrete clinical entities, we could consider HARs as overlapping genetic signals that disrupt common biological mechanisms. These biological mechanisms would converge in neurodevelopmental pathways and involve processes guiding neural proliferation, differentiation, architectural organization, and functioning. Supporting this view, there are results showing that among human diseases, HAR genes are mostly associated with cognitive disorders and nervous system diseases [104].

All these data can be integrated under the umbrella of the human brain evolution hypotheses that postulate that the larger human neocortex could arise from an increasing number of cortical neurons generated in the germinal zones during foetal development [35,37,105,106]. This neocortical expansion would be driven by a greater and prolonged proliferative capacity rather than due to the differentiation capacity of the human neural stem and progenitor cells, which are differences indicative of a species-specific transcriptomic regulation of neocortex development [37,107]. In this sense, findings regarding the interaction between HARs and genes that are related to neuronal differentiation and proliferation—such as *PTBP2* or *PPP1R17* [52,60]—together with the specific enrichment of HAR genes in radial glia during neurodevelopment [58], are highly relevant. The radial glia is a major class of neural stem cells, common progenitors of neurons and oligodendrocytes that give rise to neurons and glial cells [108], which show several unique human features that pave the way for the human-specific expansion of the cortex [109]. Hence, if HARs somehow contribute to guiding the expression mechanisms of radial glia cells, these evolutionarily relevant genomic regions would be part of the neurodevelopmental program that renders the human neocortex expansion unique. Indeed, among the characteristics of human-specific cortical expansion—as compared to our closest relatives, the chimpanzees—there are differences in the proliferative capacity of neural progenitors during cortical development [37]. This is, in turn, what the radial unit hypothesis states: that the expansion of the cortical surface area is driven by the proliferation of neural progenitors, while the thickness is determined by the number of their neurogenic divisions [110]. Certainly, this hypothesis has been supported by the latest ENIGMA Consortium GWAS results [111], which describe different developmental mechanisms behind surface area expansion and cortical thickness increase. Then, the surface area would be influenced by genetic variants that alter gene regulatory activity in neural progenitor cells during foetal development. On top of that, surface area genetic determinants positively correlate with those of cognitive functioning and educational attainment, which thus entails the fact that the genetic background underlying these phenotypes shows evidence of bidirectional causation [111]. Moreover, radial-glia-specific SCZ polygenic risk has been related to neuroplasticity processes in the hippocampus [112], which is a brain structure with critical roles in terms of memory and associated cognitive dimensions [113,114]. All this evidence together suggests that sequence changes in HARs could display critical roles in radial glial neurodevelopmental features. Furthermore, it also opens the possibility that part of the variability in HAR regions could influence the brain and cognitive phenotypes as well as the SCZ-related brain structural changes.

Globally, the relationship between HARs and human-specific gene regulation fits with the hypothesis of “human evolution as a non-coding revolution” [115]. Moreover, it also aligns with the evidence that remarks the importance of gene expression regulatory mechanisms, rather than the genetic products themselves, in the pathophysiology of neurodevelopmental disorders [116,117,118,119]. Therefore, future studies assessing the role of HARs in mental disorders and the associated neurobiological pathways may help to jointly address two hypotheses on the origins of these disorders—both the neurodevelopmental and the evolutionary.

## 5. Future Perspectives

The increasing knowledge regarding the functions of HARs and the biological mechanisms in which they are involved opens new investigation venues. First, as we have underlined, many HARs have not been successfully validated, and while their biological functions are presumed based on motif-prediction algorithms, there is a need for new functional studies in order to deepen our understanding regarding their specific role. In this sense, the use of models closer to the biological reality, such as iPSC or brain organoids that can model corticogenesis, could be beneficial not only to validate HARs’ function in human neurodevelopment, but also to investigate the neurodevelopmental ontogenetic differences in patients suffering from neurodevelopmental-related psychiatric disorders. Second, to gain insights into the pathogenesis of neurodevelopmental disorders, especially with respect to SCZ or ASD, HARs’ functional data should be combined with results on rare genetic variants, such as those coming from whole-genome sequencing approaches. Therefore, the prioritisation and interpretation strategies in whole-genome sequencing approaches should consider not only exonic or promoter variants, but also regulatory regions such as HARs. Third, it should be considered that more than 30% of the approved drugs, especially those for neurological disorders, target at least one HAR gene [104]. This opens novel investigation possibilities and puts HARs in the spotlight for developing novel therapeutic strategies. Notwithstanding, HARs should not be the only evolutionary relevant markers being inspected in the context of human-specific neurodevelopment and psychiatric susceptibility [58,120,121,122,123]. Similarly, in order to further understand the role of HARs in the architectural and information processing basis of the human brain, new structural and fMRI studies to evaluate whether HARs’ genomic variability also influences specific cognitive and psychopathological processes dominated by the DMN and the frontoparietal network should be encouraged.

## 6. Conclusions

The human brain responds adaptively and exhibits sophisticated control, purposeful behaviour, and complex cognitive abilities due to the neurodevelopmental processes, which have evolved such that they guarantee a brain architecture supporting neural integrations to, in turn, sustain these abilities. During this evolutionary process, HARs have emerged as genomic regions that have accumulated human-specific genetic changes. In this review, we have compiled the data showing that HARs participate in the genomic regulatory machinery of genes that are related to neural proliferation, differentiation, and axonogenesis, which converge in neurodevelopmental pathways. In addition, we have shown that human-specific changes in HARs endow this neurodevelopmental machinery with unique characteristics. Moreover, studies converge into the idea that HARs underlie, to a certain extent, human-specific cortical expansion, and neural integration, especially with respect to the regions within higher-order functional networks.

**Figure 2:**
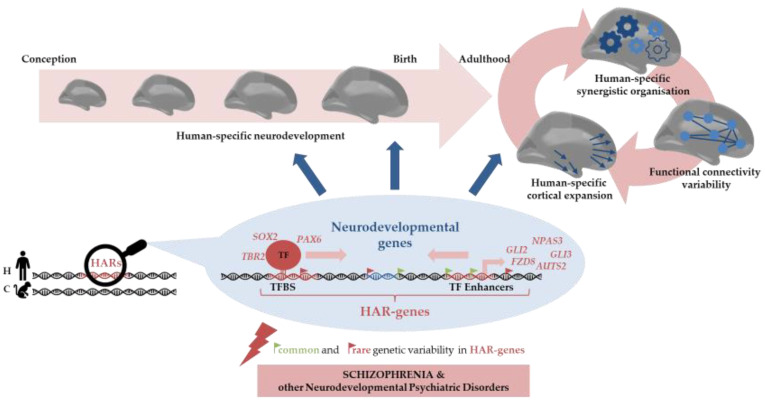
Graphical summary of Human Accelerated Regions’ (HARs) impact on neurodevelopment, brain configuration, and associated psychiatric disorders. Middle panel: HARs are evolutionarily conserved genomic regions across mammals’ evolution that accumulated human-specific (H) changes since the divergence from chimpanzees (C). Molecular studies evidence that HARs function as transcription factor binding sites (TFBS) or transcription factors (TF) of genes involved in neurodevelopmental pathways [52,58,59,60]. Top panel: The key roles of HARs in the neurodevelopmental gene regulatory machinery underlie the human-specific neurodevelopmental characteristics sustaining human-specific brain architecture, information processing, and variability in neural integration, brain traits, which in turn are associated with different psychiatric disorders [66,67,68]. Bottom panel: Common and rare genetic variability in HARs and HAR genes is related to changes in the neurodevelopmental trajectories, and therefore, sustains the inherent human variability in cognition, intelligence, behaviour, and sociability traits [66,69], but also the neural dysfunction observed in the neurodevelopmental disorder continuum. Additionally, the described effect of HAR genomic variability on the genetic determinants underlying schizophrenia and other neurodevelopmental psychiatric disorders [52,58,66,69,71,73] strengthens the paramount role of HARs in the neurodevelopmental machinery.

Despite the tight neurodevelopmental control needed that is required for proper brain function, there is still room for not only the individual differences in neurodevelopmental trajectories—which, in turn, sustain the inherent variability in cognition, intelligence, behaviour, and sociability traits found in humans—but also the eventual dysfunctions leading to neurodevelopmental disorders. As such, in this review, we have shown that HARs modulate individual differences in the context of brain functioning, but also that common and rare genetic variability in HARs is associated with neurodevelopmental psychiatric disorders, such as SCZ and ASD (Figure 2).

Thus, HARs may be the genomic regions that deserve further investigation to bridge the gap between the neurodevelopmental and evolutionary aetiological hypothesis of human-specific disorders such as schizophrenia. Future research into the molecular mechanisms that are associated with HARs will help us to understand the evolutionary changes underlying brain configuration as well as the susceptibility to human brain disorders. Achieving this will most likely result in fundamental effects on the disciplines of neuroscience and biomedicine.

## Figures and Tables

**Table 1:** Summary of the main findings of whole-genome-based studies assessing the role of HARs in neurodevelopment.

Main Objective (Methodology)	Main Results
Doan et al. [52] To characterise the functions of HARs in neurodevelopment (in silico analyses)	HARs are enriched in regulatory elements of neurodevelopmental processes in adult and foetal brain samples. Comparative genomics show HARs’ human-specific role during cortical development by altering the sequence of transcription factor motifs.
Won et al. [58] To map and characterise HAR expression patterns, tissue, and cell specificity (in silico analyses)	HARs are enriched in putative regulatory elements active prenatally and in enhancers accessible in cortex neurogenic zones. HAR genes regulate corticogenesis and cortical lamination, are upregulated during neurogenesis, and are related to outer radial glia in the developing cortex and astrocytes in adult prefrontal cortex.
Uerbbing et al. [59] To study the effect of HAR variability in human neurogenesis (massively parallel reporter assay (MPRA) in human neural stem cells)	Human-specific substitutions within HARs interact with each other and with background sequences to modify enhancer activity and modify transcription factor binding sites.
Girskis et al. [60] To study HARs’ effect on the recent evolution of the human cerebral cortex (Capture MPRAs in human neural stem cells and neurospheres)	Half of HARs act as brain enhancers, preferentially in neurodevelopment, with critical roles in corticogenesis. Most of the HARs with enhancer activity show increased enhancer activity in humans vs. chimpanzees.

**Table 2:** Summarised results of the functional validation of HAR regulatory activity on candidate genes.

HAR	Gene—Function	Validation Methodology	Main Results
**HAR1** [47]	***HAR1F* and *HAR1R*** (*Highly Accelerated Region 1A* and *1B*, 20q13.33) Non-Protein-Coding RNAs. Unknown function.	Expression assay on human embryonic and adult brain. Comparative expression analysis in embryonic macaque, mouse, and human brains	HAR1 is part of two RNA genes: *HAR1F* and *HAR1R,* both expressed in the developing cortex and in the adult frontal cortex, hippocampus, thalamus, and hypothalamus. *HAR1R* is expressed in an attenuated way compared to *HAR1F*. There are differences in *HAR1F* and *HAR1R* expression ratios between humans and mice.
**HACN96, HAR202, 2xHAR142, HAR89, 2xHAR223, 2xHAR157, 2xHAR122, HAR96, HACNS658, HAR189, HACNS553, HAR21, HACNS221, HAR173** [62,63]	***NPAS3*** (*Neuronal PAS Domain Protein 3*, 14q13.1) Transcription factor involved in the control of neurosignalling pathways during neurogenesis.	Expression assays in transgenic zebrafish and mice. Comparative expression of human, chimpanzee, and mouse HAR orthologs. Hybridisation in transgenic mice	Most HARs (11/14) in *NPAS3* act as transcriptional enhancers during brain development (in transgenic zebrafish and mice), and all human-specific substitutions produce gain or loss of transcription factor binding sites. Human HARs have different expression patterns in location and intensity compared to chimpanzee and mouse orthologs. *NPAS3* and 2xHAR142 expression patterns overlap in the forebrain regions with neural progenitor cells in transgenic mice.
**HAR31, HACNS174, HACNS369** [64]	***AUTS2*** (*Autism Susceptibility Candidate 2*, 7q11.22) Transcription factor involved in neurodevelopmental regulation, axon and dendrite elongation, and neuronal migration.	Targeted expression assays in transgenic zebrafish and mice	HAR31 and HAR369 show regulatory effects on *AUTS2* expression in the brain of zebrafish and mice.
**HARE5** [65]	***FZD8*** (*Frizzled Class Receptor 8*, 10p11.21) Receptor in the WNT pathway implicated in cortical development.	Comparative expression of human and chimpanzee HAR orthologs in transgenic mice	Human-specific changes in HARE5 result in different transcription factor binding sites and drive an earlier and more robust brain expression of *FZD8* at the onset of corticogenesis.
**HAR-HSTR1** [61]	***HSTR1*** (*Human-Specific Tandem Repeat 1*, 20p) Non-Protein-Coding RNA. Unknown function. Non-annotated gene.	Targeted expression assays in HEK293T cells. Comparative expression of human, chimpanzee, gorilla, and orangutan HAR orthologs through luciferase reporter assays	This HAR-*HSTR1* acts as the promoter of the *HSTR1* gene. Human HAR sequence has higher promoter activity than the other primate orthologs.
**HAR426** [52]	***CUX1*** (*Cut Like Homeobox 1*, 7q22.1) Transcription factor involved in the control of neuronal differentiation.	Mutant and wild-type HAR mutation effect through luciferase reporter assays in mouse neural-precursor-like cells	A rare mutation in HAR426 is associated with a three-fold increased *CUX1* enhancer activity and promoter expression.
**HAR169** [52]	***PTBP2*** (*Polypyrimidine Tract Binding Protein 2*, 1p21.3) RNA-binding protein and brain-specific splicing regulator essential for neuronal differentiation.	Mutant and wild-type HAR mutation effect through luciferase reporter assays in mouse neural-precursor-like cells. Massively parallel reporter assays in mouse neurospheres	The mutation in HAR169 alters transcription factor binding sites and causes a 50% reduction in *PTBP2* enhancer activity in neural-like mouse cells. The mutation in HAR169 produces a 40% reduction in *PTBP2* enhancer activity in mouse neurospheres.
**HAR1325** [52]	***GPC4*** (*Glypican Proteoglycan 4*, Xq26.2) Protein essential for excitatory synapse development in mice and dosage-sensitive gene in adult human brain.	Mutant and wild-type HAR mutation effect through luciferase reporter assays in mouse neural-precursor-like cells. Massively parallel reporter assays in mouse neurospheres	Two rare mutations in HAR1325 alter transcription factor binding sites and cause a 20–30% reduction in the *GPC4* enhancer activity in neural-like mouse cells. One of the mutations in HAR1325 produces a 30% reduction in *GPC4* enhancer activity in mouse neurospheres.
**HAR4** [58]	***GLI2*** (*GLI family zinc finger 2*, 2q14.2) Transcription factor in the Sonic Hedgehog (Shh) pathway critical for neural tube formation. Involved in cell growth and specialisation.	Targeted expression in primary human neural progenitor cells	Compared to *GLI2* expression without HAR4, the presence of HAR4 increases by 60% the expression of *GLI2* in primary human neural progenitor cells.
**HAR1225** [58]	***GLI3*** (*GLI family zinc finger 3*, 7p14.1) Transcription factor in the Shh pathway critical for neural tube formation. Essential for dorsal–ventral patterning of telencephalon and cortex formation in humans.	Targeted expression in primary human neural progenitor cells	Compared to *GLI3* expression without HAR1225, the presence of HAR1225 increases between 30–40% the expression of *GLI3* in primary human neural progenitor cells.
**HAR342** [58]	***TBR1*** (*T-Box Brain Transcription Factor 1*, 2q24.2) Transcriptional factor repressor involved in neuronal migration, laminar and areal identity, and axonal projection.	Targeted expression in primary human neural progenitor cells	Inconclusive results for HAR342’s effect on *TBR1* expression.
**HAR2635, HAR2636** [60]	***PPP1R17*** (*Protein Phosphatase 1 Regulatory Subunit 17*, 7p14.3) Phosphatase inhibitor involved in neural progenitor cell proliferation and expression regulation in the developing human cortex.	Targeted chromatin conformation capture 3C interaction analysis	HAR2635 and HAR2636 interact with promoter of *PPP1R17* in cultured neural cells, suggesting the regulating role of these HARs.

**Table 3:** Summarised findings of the studies assessing the role of HARs in brain and cognitive phenotypes.

Main Objective	Main Methodology	Main Results
Wei et al. [66] To study the evolutionary genetics of cortical expansion using HAR gene expression	Correlation analyses of HAR genes expression with cortical expansion differences from human vs. chimpanzee (sMRI data) and human vs. primates comparative gene expression. Association analyses of HAR and HAR brain genes with DMN variability (resting state fMRI data), intelligence, and sociability (based on GWAS data)	The expression profiles of HAR genes and HAR-brain genes correlate with human cortical expansion (more expansion, more expression). The highest HAR genes and HAR brain genes expression is observed at the DMN. Humans display an upregulated HAR brain gene expression in cognitive networks compared to primates. HAR brain genes modulate the DMN individual variability. HAR genes and HAR brain genes are associated with individual intelligence variability and sociability.
Li et al. [67] To study the evolutionary genetics of brain connectivity using HAR brain gene expression	Correlation analyses of HAR brain gene expression with functional connectivity data (resting state fMRI data)	HAR brain gene expression positively correlates with functional connectivity variability. HAR brain gene expression is higher in limbic, default mode, dorsal, and ventral attentional and frontoparietal networks. The most correlated genes are involved in synapse development, neurogenesis, and neuron differentiation.
Luppi et al. [68] To study the evolutionary genetics of redundant and synergistic information organization using HAR brain gene expression	Correlation analyses of HAR brain gene expression with the spatial distribution of synergistic and redundant brain interactions (resting state fMRI data)	Redundant interactions predominate in the somatomotor, salience subnetworks, and visual regions, while synergy predominates in higher-order association cortices affiliated with the DMN, frontoparietal network, and limbic subnetwork. HAR brain gene expression positively correlates with the regional distribution of synergistic interactions. Spatial variation of HAR brain gene expression explains up to 30% of the regional synergy-redundancy variance.
Cheung et al. [69] To test whether genes associated with intelligence are enriched in HARs	Enrichment analyses on HARs, brain expression, and their interaction on intelligence (based on GWAS data)	Polymorphisms in HAR genes are more likely associated with intelligence than polymorphisms in other regions. The expression of HAR genes across five developmental stages is associated with intelligence variability.

Default mode network (DMN); functional magnetic resonance imaging (fMRI); genome-wide association study (GWAS); structural magnetic resonance imaging (sMRI).

**Table 4:** Summarised findings of the studies assessing the role of HARs in psychiatric disorders.

Main Objective	Main Methodology	Main Results
**Schizophrenia**		
Xu et al. [71] To study HAR enrichment on common variability associated with SCZ	HAR enrichment analysis in SCZ (based on GWAS data) and gene co-expression network analyses	SCZ-associated loci are enriched in genes near HARs, specifically in recently evolved HARs (pHARs). pHAR gene associated with SCZ converge in GABA-related pathways, neuron differentiation, cell adhesion, plasma membrane, and cadherin-binding processes. pHAR gene associated with SCZ are hub genes in regulatory networks of the human prefrontal cortex.
Srinivassan et al. [73] To study HAR enrichment of common variability associated with SCZ	HAR enrichment analysis in SCZ (based on GWAS data)	Common genetic variants associated with SCZ are enriched in HAR regions. HAR brain genes are enriched in SCZ-associated single nucleotide polymorphisms compared to other brain genes or other HARs (not brain-specific).
Wei et al. [66] To study the association of HAR genes and HAR brain genes with SCZ	Examination of potential associations of HAR and HAR brain genes with SCZ variability (based on GWAS data)	HAR genes and HAR brain genes are associated with genetic variants in SCZ.
Cheung et al. [69] To study HAR gene enrichment on genes associated with neuropsychiatric disorders conditional to developmental gene-expression patterns	HAR gene enrichment analyses in five neuropsychiatric disorders (SCZ, BPD, ASD, MDD, and ADHD, based on GWAS data) conditional to gene expression in five developmental stages	Single nucleotide polymorphisms in HAR genes are more likely associated with the risk for SCZ and MDD than polymorphisms in other regions. HAR genes highly expressed in whole brain across development are more likely associated with the risk for SCZ.
Erady et al. [76] To investigate nORF associated with HARs in the genetic architecture of SCZ and BPD	Assess the overlap between nORF and nORF differentially expressed in SCZ and BPD and HARs. nORD and HARs overlap enrichment analyses in SCZ and BPD (based on GWAS data)	There is an overlap between nORF and HARs regions, and some of these regions are associated with differential expression in SCZ and BPD. Some of these nORF and HAR overlap regions harbour loci associated with SCZ and BPD through GWAS.
Tolosa et al. [70] To study the association of common variants in *HAR1F* gene with SCZ risk and AH in SCZ	Case–control association study (285 SCZ-spectrum disorders [221 AH and 64 no AH] and 337 HC) of *HAR1F* gene (six variants genotyped) with SCZ risk	No allelic, genotypic, or haplotypic associations are found with the risk for SCZ. A six-variant haplotype increases the risk for AH in patients (OR = 2.83).
González-Peñas et al. [72] To study the association of common and rare variants in *NPAS3* HARs with SCZ risk	Case–control association study (538 SCZ and 539 HC) of *NPAS3* gene (26 variants genotyped) with SCZ risk	None of the analysed variants are associated with SCZ at allelic, genotypic, or haplotypic level.
Bhattacharyya et al. [74,75] To assess the association of variants in HARs with SCZ and cognitive performance	Case–control association study (Discovery: 494 patients and 436 healthy controls (HC); Replication: 552 patients and 551 HC) of HARs (49 variants genotyped) with SCZ risk. Case–control association study in a subsample (331 patients and 235 HC) of HARs (49 variants genotyped) with cognition variability	Four variants are significantly associated with SCZ. Three of them interfere with transcription factor binding sites (TFBS) and have methylation marks of active promoters, repressors, or enhancers in the brain. Five variants significantly modulate cognitive performance within controls (13 variants) or patients (six variants). All these variants interfere with TFBS, and five had methylation marks of active promoters, repressors, or enhancers.
**Other neurodevelopmental psychiatric disorders and related syndromes**		
Doan et al. [52] To evaluate the mutational landscape of HARs and their contribution to ASD	HAR gene mapping through in silico chromatin interaction data. Assessment of copy number variants (CNVs) in 2100 ASD-sibs sample. Assessment of rare mutations in HARs through whole-genome sequencing in 218 ASD families	HAR genes are dosage-sensitive and enriched for associations with ASD and SCZ, especially the neurally active HAR genes. The rare de novo CNVs are more prevalent in HARs than in other genomic regions and are associated with ASD. Individuals with ASD have an excess of rare HAR mutations compared to non-affected individuals. The HARs harbouring these rare mutations are enriched for transcription factor binding sites. Among the genes flanking the rare HAR mutations, 70% are expressed in the brain and associated with ASD.
Won et al. [58] To study the role of HAR genes in the susceptibility for neurodevelopmental disorders	HAR enrichment analysis with genes harbouring loss-of-function variants in ASD, SCZ, and DD data	HAR genes are enriched for loss-of-function-intolerant genes that harbour de novo mutations associated with ASD and developmental delay.
Wei et al. [66] To study the association of HAR genes and HAR brain genes with ASD variability and brain structural changes found in psychiatric disorders	Examination of potential associations of HAR and HAR brain genes with genes associated with ASD (based on rare variants of brain disorders). Correlation analyses of HAR brain gene expression with structural alterations across psychiatric disorders (sMRI data on SCZ, BPD, ASD, MDD, OCD)	HAR genes and HAR brain genes are enriched for genes with rare variants associated with ASD compared to other regions in the genome. Changes in brain structure observed across psychiatric disorders correlate with HAR brain gene expression patterns.
Takahashi et al. [77] To identify the molecular pathways associated with delirium and test the enrichment of HAR genes	Functional enrichment analysis of HAR genes in delirium-associated genes (obtained from the toxicogenomics database)	The top networks associated with delirium include genes enriched in HAR brain genes.

Auditory hallucinations (AH); autism spectrum disorder (ASD); bipolar disorder (BPD); healthy controls (HC); major depressive disorder (MDD); novel open reading frames (nORF); obsessive-compulsive disorder (OCD); schizophrenia (SCZ). HARs defined based on conservation in non-human primates (pHARs); structural magnetic resonance imaging (sMRI).

## Data Availability

No new data were created or analysed in this study. Data sharing is not applicable to this article.
